# Nut Consumption Is Associated with Cognitive Status in Southern Italian Adults

**DOI:** 10.3390/nu17030521

**Published:** 2025-01-30

**Authors:** Justyna Godos, Francesca Giampieri, Evelyn Frias-Toral, Raynier Zambrano-Villacres, Angel Olider Rojas Vistorte, Vanessa Yélamos Torres, Maurizio Battino, Fabio Galvano, Sabrina Castellano, Giuseppe Grosso

**Affiliations:** 1 Department of Biomedical and Biotechnological Sciences, University of Catania, 95123 Catania, Italy; 2 Center for Human Nutrition and Mediterranean Foods (NUTREA), University of Catania, 95123 Catania, Italy; 3 Department of Clinical Sciences, Università Politecnica delle Marche, 60131 Ancona, Italy; 4 Research Group on Food, Nutritional Biochemistry and Health, Universidad Europea del Atlántico, Isabel Torres 21, 39011 Santander, Spain; 5 International Research Center for Food Nutrition and Safety, Jiangsu University, Zhenjiang 212013, China; 6 Joint Laboratory on Food Science, Nutrition, and Intelligent Processing of Foods, Polytechnic University of Marche, Italy, Universidad Europea del Atlántico Spain and Jiangsu University, China at Polytechnic University of Marche, 60130 Ancona, Italy; 7 School of Medicine, Universidad Católica de Santiago de Guayaquil, Av. Pdte. Carlos Julio Arosemena Tola, Guayaquil 090615, Ecuador; 8 Escuela de Nutrición y Dietética, Universidad Espíritu Santo, Samborondón 0901952, Ecuador; 9 Universidad Internacional Iberoamericana, Arecibo, PR 00613, USA; 10 Universidad Internacional do Cuanza, Cuito EN250, Bié, Angola; 11 Universidad Internacional Iberoamericana, Campeche 24560, México; 12 Universidad de La Romana, La Romana 22000, Dominican Republic; 13 International Joint Research Laboratory of Intelligent Agriculture and Agri-Products Processing, Jiangsu University, Zhenjiang 212013, China; 14 Department of Educational Sciences, University of Catania, 95124 Catania, Italy

**Keywords:** nuts, cognitive, Mediterranean diet

## Abstract

Background: Nut consumption has been considered a potential protective factor against cognitive decline. The aim of this study was to test whether higher total and specific nut intake was associated with better cognitive status in a sample of older Italian adults. Methods: A cross-sectional analysis on 883 older adults (>50 y) was conducted. A 110-item food frequency questionnaire was used to collect information on the consumption of various types of nuts. The Short Portable Mental Status Questionnaire was used to assess cognitive status. Multivariate logistic regression analyses were performed to calculate odds ratios (ORs) and 95% confidence intervals (CIs) for the association between nut intake and cognitive status after adjusting for potential confounding factors. Results: The median intake of total nuts was 11.7 g/day and served as a cut-off to categorize low and high consumers (mean intake 4.3 g/day vs. 39.7 g/day, respectively). Higher total nut intake was significantly associated with a lower prevalence of impaired cognitive status among older individuals (OR = 0.35, CI 95%: 0.15, 0.84) after adjusting for potential confounding factors. Notably, this association remained significant after additional adjustment for adherence to the Mediterranean dietary pattern as an indicator of diet quality, (OR = 0.32, CI 95%: 0.13, 0.77). No significant associations were found between cognitive status and specific types of nuts. Conclusions: Habitual nut intake is associated with better cognitive status in older adults.

## 1. Introduction

The largest share of non-communicable disease prevalence and mortality in developed countries is currently attributed to cardiometabolic diseases [1]. However, the increasing life expectancy has also led to a rise in other common age-related conditions, such as cognitive decline and subsequent pathological neurodegenerative diseases [2]. Dementia is characterized by progressive cognitive impairment in several domains, including memory, learning, language, and orientation. This dysfunction may begin as mild cognitive impairment (MCI) and, depending on its pathophysiology, can progress to dementia-related disorders such as vascular dementia or Alzheimer’s disease (AD) [3,4]. The global prevalence of cognitive disorders is rising, with dementia currently one of the major causes of disability among older individuals, affecting over 55 million people worldwide [5]. Given the growing impact of these conditions in an aging population that is otherwise often free of cardiometabolic diseases, it is of paramount importance to identify successful strategies to prevent the deterioration of health and quality of life in older adults.

In the past few years, scientific interest has focused on the relationship between diet and brain function [6]. Among the most studied prevention strategies to reduce the incidence of neurodegenerative diseases, adopting healthy dietary patterns has shown promising associations with cognitive health [7]. Oxidative stress and neuroinflammation have emerged as potential targets for modulating brain function and reducing the risk of cognitive disorders [8]. Diets rich in plant-derived food products, including vegetables, fruits, legumes, olive oil, and nuts, have been associated with an increased intake of nutrients, such as healthy fats, antioxidant vitamins, and phytochemicals [9]. These nutrients have been demonstrated to positively impact intestinal integrity [10] and gut microbiota toward an anti-inflammatory profile and may exert direct effects on the brain, promoting vascular health, counteracting neurodegeneration, and improving cognitive function [11]. While most studies explore the effects of broad dietary patterns, it is crucial to identify specific dietary components that are independently associated with cognitive outcomes.

Among the individual components of healthy dietary patterns, such as the Mediterranean diet [12,13], nuts have been studied for their unique nutritional profile, which may provide a credible rationale for potential positive effects on human brain health [14,15]. Tree nuts are a type of dry fruit that contain an edible seed and a hard shell, typically consumed as part of the traditional Mediterranean diet [12]. This group of nuts generally include pecans (*Carya illinoiensis*), almonds (*Prunus amigdalis*), cashews (*Anacardium occidentale*), hazelnuts (*Corylus avellana*), macadamias (*Macadamia integrifolia*), walnuts (*Juglans regia*), Brazil nuts (*Bertholletia excelsa*), chestnuts (*Castanea sativa*), pistachios (*Pistacia vera*), and pine nuts (*Pinus pinea*). Although technically classified as groundnuts, peanuts (*Arachis hypogaea*) are also frequently included in research on nuts [15]. Despite differences across types, nuts are generally rich in mono- and poly-unsaturated fatty acids (MUFA and PUFA, respectively), fiber, vitamins, and (poly)phenols, all of which have demonstrated potential benefits in aging individuals [16]. Moreover, there is evidence that nut consumption is associated with improvements in cardiometabolic parameters, including a decrease in total cholesterol and LDL cholesterol [17], and a lower risk of cardiovascular disease [18]. These findings suggest a potential role in preventing vascular dementia and cognitive decline. Evidence on the association between nut consumption and cognitive outcomes is growing, although the majority of studies have focused on walnuts and yielded mixed results [19]. Interestingly, recent studies showed that increased adherence to the Mediterranean diet in the Italian population would lead to higher adequacy to national dietary guidelines, although there is no clear mention of nut intake [20,21]. This study aimed to comprehensively evaluate the relationship between nut consumption and cognitive status.

## 2. Materials and Methods

### 2.1. Study Population

This cross-sectional study utilized a subset of participants from the Mediterranean Healthy Eating, Aging, and Lifestyles (MEAL) study, an observational investigation aimed at exploring the association between Mediterranean dietary and lifestyle habits and the prevalence of chronic diseases among Mediterranean populations. A comprehensive description of the MEAL study methodology has been published previously [22]. The study cohort, drawn from medical records of general practitioners in Catania, a major urban center on Sicily’s eastern coast, southern Italy, included men and women aged 18 and older who were randomly selected between 2014 and 2015. Sampling implied stratifying participants by age, sex, and residential area before randomly assigning them to subgroups. General practitioners acted as primary sampling units, while their registered patients represented the final study sample. Pregnant women were excluded from participation. The recruitment process achieved an 85% response rate, yielding a final sample of 2044 respondents from an initial invitation list of 2405. This analysis specifically focused on participants aged 50 years or older who had complete dietary intake data (n = 883). All procedures in the study followed the World Medical Association’s Declaration of Helsinki (1989).

### 2.2. Dietary Habits

Dietary intake was evaluated through a Food Frequency Questionnaire (FFQ), specially developed and validated for the Sicilian population [23,24]. The FFQ covered 110 food and beverage items reflecting the typical intake over the past six months. Participants were requested to report their regular frequency of consumption for each item, with nine response options ranging from “never” to “4–5 times per day”. Seasonal food items were adjusted based on their availability and annual consumption frequency. Dietary intakes considered unreliable (i.e., <1000 or >6000 kcal/day), identified through case-by-case verification, were excluded from the analysis.

Total nut consumption was calculated as a sum of consumption of each type of nut, such as chestnuts, peanuts, pistachios, walnuts, almonds, and hazelnuts, based on their frequency of consumption. Total nut or individual nut consumption did not comprise the use of nut-based spreads.

Adherence to the Mediterranean diet was assessed through the application of a literature-based index applying a scoring according to higher consumption of foods in line (fruit, vegetables, legumes, grains, fish, and olive oil), encouraged for moderation (alcohol) or limited (dairy and meat) consumption in the context of the Mediterranean diet [25]. Notably, this score does not take into account the consumption of nuts, hence these variables could not be related (to avoid the confounding effects of the overall diet).

### 2.3. Cognitive Status Assessment

Cognitive status was evaluated with the Short Portable Mental Status Questionnaire (SPMSQ) [26], a validated tool for evaluating cognitive impairment in general and clinical populations [27], formerly used in Italian studies [28]. This 10-item questionnaire, administered by a clinician in-office or in a hospital setting, categorized cognitive status as follows: (i) intact (fewer than 3 errors), (ii) mild impairment (3 to 4 errors), (iii) moderate impairment (5 to 7 errors), and (iv) severe impairment (8 or more errors). In this report, cognitive impairment was defined by a threshold of three or more errors.

### 2.4. Statistical Analysis

Categorical variables were summarized as absolute frequencies and percentages, whereas continuous variables were reported as means and standard deviations. Participants were categorized based on nut consumption (never vs. ever) to examine differences in baseline characteristics. Associations between nut consumption and dietary variables (e.g., total energy, macronutrients, micronutrients, and total polyphenol intake) were analyzed by comparing means and standard deviations. Statistical differences were assessed using Chi-square tests for categorical variables, Student’s *t*-tests were applied for continuous variables with a normal distribution, and Mann–Whitney U-tests for non-normally distributed variables. Multivariate logistic regression models, adjusted for energy, were employed to estimate odds ratios (ORs) and 95% confidence intervals (CIs) for the associations between nut intake (total and specific types) and cognitive status. Additional multivariate models, adjusted for variables such as age, sex, education, occupation, smoking, physical activity level, and body mass index, were conducted to determine the independence of associations. To further explore dietary quality, a final model adjusted for adherence to the Mediterranean diet was included. All tests were two-sided with a significance level of 5%. Statistical analyses were conducted using SPSS software, version 29 (SPSS Inc., Chicago, IL, USA).

## 3. Results

The descriptive characteristics of the 883 older individuals included in this study are shown in Table 1. The mean total nut intake in the consumption group was 22.3 (SD 32.6) g/d, an average of almost one portion of nuts. When consumed, chestnuts are the nuts with the highest weight (16.2 ± 45.0 g/d). Among the others, the largest contributors to total nuts are walnuts (2.1 ± 4.5 g/d) and almonds (2.1 ± 4.2 g/d), followed by hazelnuts (1.2 ± 3.6 g/d), peanuts (1.2 ± 2.6 g/d), and pistachios (0.5 ± 1.1 g/d), reflecting a sporadic consumption of a portion per week. Regarding demographic characteristics, individuals in the consumption group were younger (*p* = 0.006). Also, significant differences were found in smoking and occupational status (*p* < 0.001, *p* = 0.033, respectively), with a higher percentage of former smokers in the low-consumption group and a lower percentage of unemployed individuals. There were no significant differences observed in sex distribution, educational status, physical activity level, and BMI. Although marginal in terms of score, a significant difference in adherence to the Mediterranean dietary pattern between the two groups of nut consumption was also found (Table 1).

Table 2 presents the differences in daily total energy, macro- and micronutrients, and total polyphenol intake between low- and high-consumption groups. The groups had no significant difference in the total daily energy intake. In terms of macronutrient intake, individuals in the high-consumption group had significantly higher intake of total fat (*p* = 0.016), trans fatty acids (*p* = 0.031), MUFA (*p* = 0.039), and omega-6 fatty acids (*p* = 0.004), but lower intake of total carbohydrates (*p* = 0.011). The individuals in the high-consumption group were also characterized by higher intakes of all explored vitamins, including vitamins A, C, E, D, and B12, and total (poly)phenols (*p* < 0.001). Additionally, high nut consumers had higher intakes of potassium (*p* < 0.001), iron (*p* = 0.002), magnesium (*p* = 0.017), and zinc (*p* = 0.019), but lower selenium intake (*p* = 0.003), compared to those in the low-consumption group.

The multivariate logistic regression models examining the association between total nut intake, specific nut types, and cognitive status are presented in Table 3. Higher total nut intake was significantly associated with a lower prevalence of impaired cognitive status among older individuals (model 2, OR = 0.35, CI 95%: 0.15, 0.84) after adjusting for potential confounding factors. Notably, this association remained significant after additional adjustment for adherence to the Mediterranean dietary pattern as an indicator of diet quality (model 3, OR = 0.32, CI 95%: 0.13, 0.77). No significant associations were found between cognitive status and specific types of nuts.

## 4. Discussion

This study investigated the association between nut consumption and cognitive status among a sample of middle-aged and older adults. The results indicated a significant association between total nut consumption and a reduced likelihood of cognitive impairment. However, some discrepancies were observed regarding which specific types of nuts were most strongly associated with putative cognitive benefits.

A summary of existing evidence on the relationship between nut consumption and cognitive function suggests that, although the results from observational and intervention studies are not entirely consistent, nut consumption may have favorable effects on cognition, especially in populations at higher risk of cognitive decline [29]. Observational studies conducted to test the association between habitual nut intake and cognitive outcomes have reported various significant results, including associations between more frequent nut consumption and slower cognitive decline [30,31], better cognitive status [32], and a reduced risk of mild cognitive impairment [33,34]. Several intervention studies have also been performed, yielding quite consistent results. In a crossover study, a mixed-nut intervention resulted in improvements in brain vascular function and memory [35]. In another crossover study, administering 30 g/day of mixed nuts for 28 days resulted in a significant improvement in cognitive performance [36]. Finally, a randomized clinical trial showed that peanut intake, in the form of roasted peanuts and peanut butter, was associated with improved cognitive performance [37].

From a pathophysiological point of view, early disruptions of the blood–brain barrier and neurovascular dysfunction are key factors in the pathogenesis of both vascular cognitive impairment and Alzheimer’s disease [38,39]. Additionally, beta-amyloid is the primary component of amyloid plaques, which characterize the anatomopathological changes observed in the brains of Alzheimer’s disease patients [40]. Preclinical studies have hypothesized that nut consumption may reduce oxidative stress and prevent cell death caused by beta-amyloid protein deposition, as well as play a protective role in endothelial health [41].

Incorporating nuts into dietary patterns could increase the intake of healthy MUFA and PUFA. The fat profile of each nut varies as follows: almonds, hazelnuts, peanuts, and pistachios are higher in MUFAs, while walnuts are notable for containing alpha-linoleic acid (ALA), an omega-3 PUFA, with smaller amounts also found in hazelnuts. Both MUFAs and PUFAs have been suggested to potentially benefit the aging brain [42]. Omega-3 PUFA, particularly DHA and EPA, play a crucial role in preventing cognitive decline through a variety of interconnected mechanisms. DHA, as a key structural component of neuronal membranes, enhances membrane fluidity and synaptic plasticity, which are essential for efficient neurotransmission and cognitive function [43]. This structural role of DHA also facilitates optimal receptor function and signaling in the brain [44]. Preclinical evidence indicates a promising role for these fats in preventing or counteracting neuroinflammation, which may chronically contribute to the development of neurodegenerative diseases [45]. In fact, both DHA and EPA possess anti-inflammatory properties, which are critical for mitigating chronic neuroinflammation occurring not only in pathological statuses but also in aging brains [46]. They help modulate the activation of microglia, the brain’s resident immune cells, and reduce the production of pro-inflammatory cytokines [47]. Additionally, EPA is converted into specialized pro-resolving mediators, such as resolvins and protectins, which actively resolve inflammation [47]. Both DHA and EPA also function as antioxidants, reducing oxidative stress by neutralizing reactive oxygen species and enhancing the expression of antioxidant enzymes, thereby protecting neurons from oxidative damage [48]. Furthermore, DHA promotes the expression of the brain-derived neurotrophic factor (BDNF), a key protein involved in neuronal survival, synaptic plasticity, and learning, which helps counteract age-related neuronal loss [49]. Moreover, healthy fats have been described to improve vascular and endothelial health, potentially mitigating early mechanisms of brain pathology, neurodegeneration, and subsequent cognitive impairment [50]. They further support brain health by promoting neurogenesis, especially in the hippocampus, a region critical for memory and learning, and by improving cerebral blood flow through enhanced endothelial function and nitric oxide production, ensuring adequate nutrient and oxygen supply to brain cells [51,52]. 

Nuts are also a source of (poly)phenols, with phenolic acids (particularly hydroxybenzoic acids) being among the most common. Although most evidence from observational studies relies on flavonoid intake and its relationship with cognitive decline [53], a few studies have demonstrated that higher dietary intakes of phenolic acids are associated with improved cognitive outcomes [54,55]. These molecules, especially their metabolites have been demonstrated to pass through the blood–brain barrier and support neuronal health by inhibiting various inflammatory pathways, such as the nuclear factor erythroid 2–related factor 2 (Nrf2), nuclear factor kappa-light-chain-enhancer of activated B cells (NF-κB), signal transducer and activator of transcription 3 (STAT3), and mitogen-activated protein kinase (MAPK). They also promote the function of the antioxidant enzymes glutathione peroxidase (GPx), catalase (CAT), and antioxidants-related superoxide dismutase (SOD) [56,57]. While a degree of skepticism rises when considering the actual bioavailability of (poly)phenols, emerging preclinical evidence suggests that phenolic acids may reduce the release of pro-inflammatory cytokines (such as, TNF-α, IL-6, IL-1β, and TGF-β), inhibit amyloid beta accumulation, and mitigate memory deficits [58]. Moreover, some studies also hypothesize that phenolic acids are involved in neurotransmission, including the suppression of monoamine oxidases (MAO-A and MAO-B) and catechol-O-methyltransferase (COMT) enzymes [59].

Nut consumption has also been investigated for its potential role in promoting human health through modulation of the gut microbiota [60]. Only a minority of molecules are taken up in the small intestine, while the majority of (poly)phenols make their way to the colon, where they are metabolized by the gut microbiota [61,62]. The main microbial-derived metabolites include 4-hydroxybenzoic acid, protocatechuic acid, gallic acid, vanillic acid, and syringic acid, all of which have been widely studied for their antioxidant properties [63,64]. Additionally, the phytochemical and fiber content of nuts may promote a prebiotic effect, leading to an alteration of gut microbiota composition [65]. A recent review of trials aiming to investigate the effects of nut supplementation on gut microbiota composition reported an increase in the proportion of *Clostridium*, *Dialister*, *Lachnospira*, and *Roseburia*, along with a significant decrease in Parabacteroides following nut administration. However, no effects were recorded on bacterial phyla, overall diversity, or stool output [66]. These genera are known to produce butyrate, an important energy source for intestinal colonocytes and essential for maintaining the integrity of the intestinal epithelium [67,68]. Interestingly, some measures of gut microbiota diversity, a marker for gut health, also appear to depend on the type of nut consumed [69]. Studies have shown that walnuts, almonds, and pistachios in particular, yield significant effects on microbiota diversity [70,71]. However, human trials are only recently emerging and the limited number of studies on the modulatory effects of nuts on the gut microbiota show the overall limit of current evidence [72].

The findings of this study should be considered in light of some limitations. First, cross-sectional analysis allows for the identification of observational associations but does not permit the assessment of cause-and-effect relationships. Second, dietary intakes were self-reported, hence potentially introducing recall bias. Third, cognitive status was measured using a screening tool, thus it does not necessarily reflect pathological conditions. Additionally, there is potential confounding from an overall healthier lifestyle typically associated with higher nut intake. As already stated in previous studies, nut consumption tends to cluster with health-promoting behaviors (e.g., higher levels of physical activity, non-smoking, etc.) as well as with a more health-conscious mindset, and higher educational attainment [73]. Moreover, other factors not necessarily associated with nut consumption may still play a role in the cognitive status in the elderly (i.e., social engagement) but remain unexplored in the present study. Finally, only a limited number of individuals never consumed nuts, resulting in an unbalanced numerosity between groups (nut consumption is fairly common in Mediterranean populations); although the distribution of variables and the resulting confidence intervals do not suggest any bias, we cannot exclude that such a grouping may still have affected the results. Moreover, we are not aware of the true effects of nut consumption over time (sporadic larger portions vs. frequent smaller portions) as well as the actual preference of consumption in the population, hence the results from this study better refer to rank the sample (i.e., consumers vs. non-consumers) rather than identify the exact amount of nuts to exert positive effects on the brain.

## 5. Conclusions

In conclusion, nut consumption was associated with better cognitive status in southern Italian adults. However, no specific nut type demonstrated an individual association with cognitive status, suggesting that the potential brain health benefits may arise from overall nut consumption rather than a particular type. Additional longitudinal research would provide more robust evidence regarding the temporal relationship between nut consumption and cognitive decline, thereby strengthening the findings of this study.

## Figures and Tables

**Table 1:** Demographic characteristics of the study participants according to nut consumption (n = 883).

	Nut Consumption		
	Never (n = 66)	Ever (n = 817)	*p*-Value
Age, mean (SD)	68 (9.41)	65 (9.53)	0.006
Sex, n (%)			0.886
Men	28 (42.4)	354 (43.3)	
Women	38 (57.6)	463 (56.7)	
Smoking status, n (%)			<0.001
Never	36 (54.5)	461 (56.4)	
Current	4 (6.1)	183 (22.4)	
Former	26 (39.4)	173 (21.2)	
Occupational level, n (%)			0.033
Unemployed	8 (12.3)	195 (27.5)	
Low	17 (26.2)	118 (16.7)	
Medium	22 (33.8)	212 (29.9)	
High	18 (27.7)	183 (25.8)	
Educational level, n (%)			0.135
Low	39 (59.1)	412 (50.4)	
Medium	14 (21.2)	271 (33.2)	
High	13 (19.7)	134 (16.4)	
Physical activity level, n (%)			0.069
Low	4 (10.5)	192 (27.0)	
Medium	24 (63.2)	346 (48.7)	
High	10 (26.3)	173 (24.3)	
BMI, mean (SD)	27 (4.69)	27 (4.27)	0.678
Mediterranean diet adherence, mean (SD)	11.9 (2.6)	12.1 (2.1)	0.023

**Table 2:** Mean (and standard deviation) consumption of micronutrients, macronutrients, and total polyphenols according to nut consumption in the study sample.

	Nut Consumption		
	Never (n = 66)	Ever (n = 817)	*p*-Value
Energy intake (kcal/d)	2056.0 (632.0)	2025.3 (611.8)	0.969
Macronutrients			
Protein (g/d)	80.4 (20.4)	84.1 (27.2)	0.279
Fat (g/d)	53.2 (15.7)	60.0 (22.4)	0.016
Cholesterol (mg/d)	177.1 (55.7)	186.9 (83.9)	0.352
Saturated fatty acids (%)	23.2 (8.8)	23.3 (9.1)	0.891
Trans fatty acid (%)	29.4 (8.5)	32.4 (10.8)	0.031
MUFA (%)	23.1 (6.3)	25.2 (8.3)	0.039
PUFA (%)	10.5 (2.1)	12.0 (4.9)	0.330
Omega-3 (%)	2.0 (0.9)	1.7 (0.9)	0.074
Omega-6 (%)	8.6 (3.2)	10.0 (3.9)	0.004
Carbohydrates (g/d)	329.6 (126.0)	295.4 (103.4)	0.011
Total fiber (g/d)	29.71 (11.3)	32.6 (13.5)	0.089
Micronutrients			
Vitamin A retinol eq (μg/d)	673.0 (229.5)	878.8 (424.0)	<0.001
Vitamin C (mg/d)	102.6 (46.5)	161.8 (94.7)	<0.001
Vitamin E (mg/d)	6.9 (2.0)	8.7 (3.1)	<0.001
Vitamin D (μg/d)	3.7 (2.1)	5.6 (5.2)	0.002
Vitamin B12 (μg/d)	4.9 (2.1)	6.1 (3.9)	0.011
Total polyphenols (mg/d)	309.2 (166.6)	660.7 (447.5)	<0.001
Minerals			
Sodium (mg/d)	2616.2 (1035.7)	2738.4 (1021.6)	0.351
Potassium (mg/d)	2991.4 (750.9)	3702.8 (1302.6)	<0.001
Iron (mg/d)	13.2 (4.0)	15.4 (5.5)	0.002
Calcium (mg/d)	748.8 (212.1)	807.3 (324.1)	0.150
Magnesium (mg/d)	358.1 (88.3)	396.4 (126.9)	0.017
Zinc (mg/d)	11.0 (3.2)	12.3 (4.3)	0.019
Selenium (μg/d)	120.2 (51.4)	103.4 (43.8)	0.003

**Table 3:** Odds ratios (ORs) and 95% confidence intervals (CIs) of the association between nut consumption and cognitive status.

	Cognitive Status, OR (95% CI)		
	Model 1 ^a^	Model 2 ^b^	Model 3 ^c^
Total nuts	0.40 (0.20, 0.80)	0.35 (0.15, 0.84)	0.32 (0.13, 0.77)
Chestnuts	0.91 (0.52, 1.59)	1.19 (0.61, 2.33)	1.14 (0.58, 2.23)
Peanuts	1.38 (0.78, 2.45)	1.16 (0.60, 2.27)	1.21 (0.62, 2.37)
Pistachios	1.43 (0.78, 2.62)	1.32 (0.64, 2.71)	1.31 (0.63, 2.72)
Walnuts	1.82 (0.91, 3.61)	2.25 (0.96, 5.23)	2.15 (0.92, 5.03)
Almonds	0.86 (0.46, 1.60)	0.89 (0.43, 1.88)	0.90 (0.43, 1,90)
Hazelnuts	0.56 (0.32, 1.10)	0.52 (0.25, 1.07)	0.52 (0.25, 1.09)

^a^ Adjusted for total energy intake; ^b^ adjusted as model 1 plus age, sex, educational and occupational status, smoking status, and physical activity level and body mass index; ^c^ adjusted as model 2 plus adherence to the Mediterranean diet.

## Data Availability

The data that support the findings of this study are available upon reasonable request.

## Abbreviations

The following abbreviations are used in this manuscript:

AD	Alzheimer’s disease
ALA	Alpha-linoleic acid
CAT	Catalase
BDNF	Brain-derived neurotrophic factor
CIs	Confidence intervals
COMT	Catechol-O-methyltransferase
FFQ	Food frequency questionnaire
GPx	Glutathione peroxidase
MAO	Monoamine oxidases
MAPK	Mitogen-activated protein kinase
MCI	Mild cognitive impairment
MUFAs	Mono-unsaturated fatty acids
NF-κB	Nuclear factor kappa-light-chain-enhancer of activated B cells
Nrf2	Nuclear factor erythroid 2–related factor 2
ORs	Odds ratios
PUFAs	Poly-unsaturated fatty acids
SOD	Superoxide dismutase
SPMSQ	Short Portable Mental Status Questionnaire
STAT3	Signal transducer and activator of transcription 3
